# Sympathetic signaling facilitates progression of neuroendocrine prostate cancer

**DOI:** 10.1038/s41420-021-00752-1

**Published:** 2021-11-22

**Authors:** Shubham Dwivedi, Maricris Bautista, Sanskriti Shrestha, Hussain Elhasasna, Tanaya Chaphekar, Frederick S. Vizeacoumar, Anand Krishnan

**Affiliations:** 1 Department of Anatomy, Physiology, and Pharmacology, College of Medicine, University of Saskatchewan, Saskatoon, SK S7N 5E5 Canada; 2 Cameco MS Neuroscience Research Centre (CMSNRC), Saskatoon, SK S7K 0M7 Canada; 3 Division of Oncology, College of Medicine, University of Saskatchewan, Saskatoon, SK S7N 5E5 Canada; 4 Department of Arts and Science, University of Tennessee, Knoxville, TN USA

**Keywords:** Prostate cancer, Cancer microenvironment

## Abstract

The progression of prostate cancer (PC) into neuroendocrine prostate cancer (NEPC) is a major challenge in treating PC. In NEPC, the PC cells undergo neuroendocrine differentiation (NED); however, the exact molecular mechanism that triggers NED is unknown. Peripheral nerves are recently shown to promote PC. However, their contribution to NEPC was not studied well. In this study, we explored whether sympathetic neurosignaling contributes to NED. We found that human prostate tumors from patients that later developed metastases and castration-resistant prostate cancer (CRPC), a stage preceding to NEPC, have high sympathetic innervations. Our work revealed that high concentrations of the sympathetic neurotransmitter norepinephrine (NE) induces NED-like changes in PC cells in vitro, evident by their characteristic cellular and molecular changes. The NE-mediated NED was effectively inhibited by the Adrβ2 blocker propranolol. Strikingly, propranolol along with castration also significantly inhibited the development and progression of NEPC in vivo in an orthotopic NEPC model. Altogether, our results indicate that the NE-Adrβ2 axis is a potential therapeutic intervention point for NEPC.

## Introduction

Prostate cancer (PC) is the second leading cancer diagnosed and the fifth leading cause of cancer-related deaths in men worldwide [1]. The mortality associated with PC often results from the development of treatment-resistant neuroendocrine PC (NEPC) [2]. In NEPC, prostate adenocarcinoma cells transdifferentiate into neuroendocrine cells [3, 4]. However, the exact mechanism of neuroendocrine differentiation (NED) is unknown and, hence, effective therapies for NEPC are currently lacking. The average survival period of NEPC patients is around 7–15 months [2].

Recent studies demonstrated that peripheral nerves promote PC [5–7]. For example, denser autonomic innervation is associated with poor prognosis of PC [5, 6]. In addition, several studies showed that the sympathetic neurotransmitter norepinephrine (NE) promotes PC migration and metastasis by activating adrenergic β-receptors (Adrβs) [8, 9]. Interestingly, a recent study demonstrated that Adrβ2 indeed promotes NED [10]. However, whether NE directly contributes to NED is not known. Understanding whether NE has any direct contribution to NED would expand potential therapeutic intervention points for NEPC spanning around NE biosynthesis, metabolism and NE-Adrβ axis. In this study, we explored whether NE has any direct potential to induce NED. Strikingly, we found that NE at supraphysiological concentrations induces the essential morphological and molecular features required for NED of PC cells. We revealed that the NE-mediated NED involves Adrβ2 signaling. Overall, our study indicates that targeting the NE-Adrβ2 axis may prevent NEPC development and progression.

## Results

### Human prostate tumors are densely innervated by sympathetic nerves

**Fig. 1:**
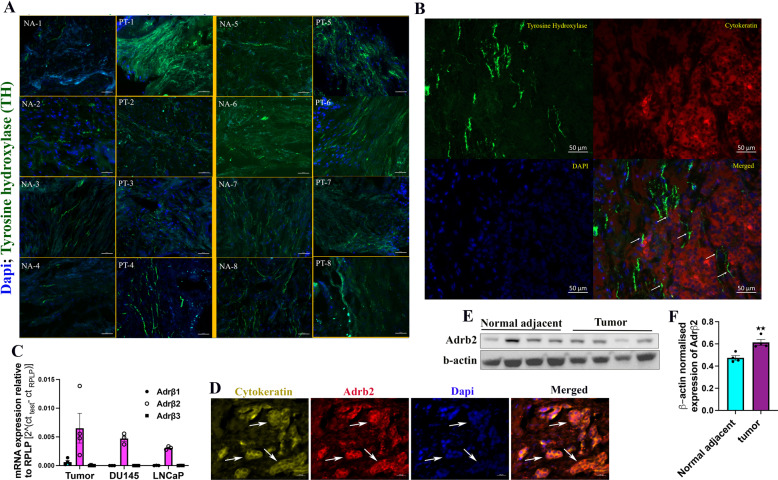
Distribution of sympathetic nerves and adrenergic receptors in human prostate tumors and corresponding normal adjacent tissues. **A** Tyrosine hydroxylase (TH: green) staining showing the distribution of sympathetic nerves in eight prostate tumors (PT) and corresponding normal adjacent tissues (NA). Dapi (blue) was used for staining the nuclei. Scale bar, 50 µm. **B** Co-staining of TH (green) and pan-cytokeratin (red; cancer cells) shows the physical contacts between sympathetic axons and cancer cells (shown using white arrows in the merged image). Scale bar, 50 µm. **C** qRT-PCR analysis of Adrβ receptor mRNAs in human prostate tumors and the PC cell lines, DU145 and LNCaP, shows relatively high expression of Adrβ2 (*n* = 4 for tumors, *n* = 3 for DU145 and LNCaP cells). **D** Immunostaining showed the expression of Adrβ2 (red) in cancer cells (yellow: cytokeratin) in human prostate tumor. The corresponding areas are shown using arrows. Scale bar, 20 µm. **E** Western blotting shows the expression of Adrβ2 in human prostate tumors and corresponding normal adjacent tissues. β-Actin was used as the loading control. **F** Quantification of “**E**” shows upregulation of Adrβ2 in human prostate tumors. The data are presented as mean ± SEM (*n* = 4) and statistically analyzed using standard Student’s *t*-test (unpaired, two-tailed). *p* < 0.05 was considered significant, where **p < 0.01 compared to the normal adjacent values.

We examined the sympathetic nerve distribution in eight human prostate adenocarcinoma and corresponding normal adjacent tissues to understand whether sympathetic axonogenesis occurs in prostate tumors. Tyrosine hydroxylase (TH) was used for staining the sympathetic nerves. Co-staining experiments with TH and the pan-neuronal marker βIII tubulin confirmed that TH specifically stains nerve fibers (Supplementary Fig. S1). We found that four out of the eight adenocarcinoma samples analyzed had higher sympathetic innervations (Fig. 1A and Table 1). The associated clinical information data from ACRB revealed that three out of those four patients that demonstrated higher sympathetic innervations in tumors had developed metastasis later. Most importantly, two of them developed castration-resistant prostate cancer (CRPC) (Table 1). Although this is a low sample size, it indicates the propensity of sympathetic signaling in facilitating PC progression. Increased tumor innervations also indicate that active axonogenesis occurs in PC. We found that the newly formed axons make specific contacts with cancer cells, indicating their direct interaction with each other (Fig. 1B). We then examined the expression of Adrβ1, Adrβ2, and Adrβ3 receptors in prostate tumors and PC cell lines and found that Adrβ2 is relatively highly expressed in them, suggesting that Adrβ2 may be the receptor mediating the sympathetic nerve–tumor interaction (Fig. 1C, D). Quantification of Adrβ2 expression in tumor samples and corresponding normal adjacent tissues revealed that Adrβ2 is overexpressed in tumors (Fig. 1E, F). Overall, our results suggest that newly formed sympathetic fibers may play critical roles in PC progression, in particular its transition to advanced stages such as CRPC, by establishing a direct interaction with cancer cells.

**Table 1:** Clinical information associated with the prostate tumors and adjacent normal tissues used for the study.

Sample number	Diagnosis	Gleason score	Metastatic occurrence	Development of CRPC within 10 years of surgery	Sympathetic innervations in adjacent normal tissues	Sympathetic innervations in tumor tissues
1	Adenocarcinoma	7	Iliac and Groin LN		2674.214	7328.8
2	Adenocarcinoma	7	Lung Lymph Node, Distant Pelvic Soft Tissue, Bone	√	3146.097	5385.674
3	Adenocarcinoma	7	Liver		6417.04	4721.599
4	Adenocarcinoma	9	Liver, Bone	√	4391.778	4930.469
5	Adenocarcinoma	7	–		5235.397	7027.38
6	Adenocarcinoma	8	–		3788.026	2950.441
7	Adenocarcinoma	7	–		6975.41	4096.858
8	Adenocarcinoma	7	–		2462.88	1823.715

### NE induces NED-like morphological changes in PC cells

**Fig. 2:**
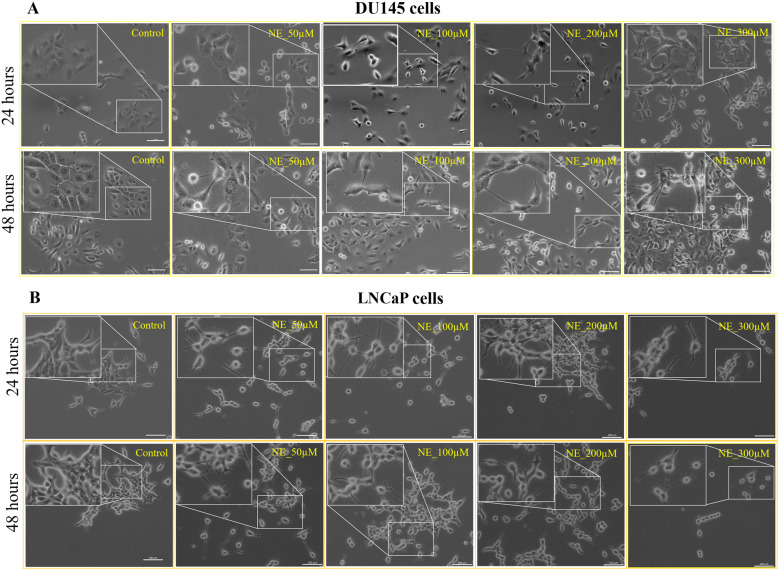
NE induces NED-like morphological changes in PC cells. **A**, **B** Dose- and time-dependent NE treatment induces NED-characteristic features, such as neurite-like extensions and compact cell bodies, in DU145 cells (**A**) and LNCaP cells (**B**). Magnified representations are provided in the insets. Scale bar, 100 µm.

Based on our observation that prostate tumors acquire higher sympathetic innervations, we asked whether the sympathetic neurotransmitter NE has any direct role in promoting PC, especially its advancement into NEPC. NED is a pre-requisite for NEPC. Therefore, we focus our studies on NE’s potential contribution to NED. We used both androgen receptor negative (AR^−^) DU145 and AR^+^ LNCaP cells, representing two spectra of PC cells, for our experiments. A previous pre-clinical study reported that early-stage prostate tumors achieve high concentrations (supraphysiological) of NE from local nerves [6]. Therefore, we considered physiologically relevant (10 µM) and supraphysiological (50 µM and above) concentrations of NE for our initial experiments. Strikingly, we observed that NE at all supraphysiological concentrations (50–300 µM) induced NED-like morphological changes in PC cells, evident from the development of neurite-like extensions and compact cell bodies (Fig. 2A, B). Although neurite-like extensions were evident in DU145 cells at 50 µM NE treatment, compact cell bodies were much apparent only from 100 µM NE onwards. At the same time, both increased neurite-like extensions and compact cell bodies appeared in LNCaP cells in response to 50 µM NE onwards. We did not find such morphological changes in PC cells at a lower concentration of NE, such as 10 µM, even after 96 h treatment (Supplementary Fig. S2). We also noted that, at a dose of 300 µM NE and above, the cells lose viability and detach from the surface (see below for additional details). Overall, our result indicates that NE induces NED-like morphological changes in PC cells, regardless of their AR status.

### NE-induced NED-like changes does not alter the viability of PC cells

We next examined the viability of NE-treated cells to determine at what supraphysiological concentrations it induces NED-like morphological changes, while retaining the cell viability intact. For this, we performed an 3-(4,5-Dimethylthiazol-2-yl)-2,5-Diphenyltetrazolium Bromide (MTT)-based cell viability assay in DU145 cells after treating them with NE in a dose- and time-dependent manner. Our assay revealed a CC_50_ value of 494, 272, 207, and 214 µM at 24, 48, 72, and 96 h NE treatment, respectively (Supplementary Fig. S3A). Combined with our previous result, it indicates that a dose range of 50–200 µM NE induces NED-like morphological changes, and at the same time, retains more than 50% cell viability for a period of 24–96 h. Our examination of propidium iodide (PI) live/dead cell staining, where the dead cells only would take up the dye, also did not show difference in PI staining between the control and up to 200 µM NE treatment over a period of 48 h, further confirming that NE at supraphysiological concentrations up to 200 µM is not cytotoxic to PC cells (Supplementary Fig. S3B). Although this result demonstrated that a single pulse of up to 200 µM NE induces NED in PC cells without affecting their viability, prostate tumors are likely to receive continuous pulses of NE from denser sympathetic innervations. Therefore, we tested the effect of frequent pulses of NE on NED by replenishing the culture media with fresh NE (50–200 µM) every 24 h for 7 days. We found NED-like morphology, such as neurite-like extensions and compact cell bodies, in these cultures at all the time points (96 and 168 h) and concentrations (50, 100, 200 µM) tested (Supplementary Fig. S4). Importantly, our PI live/dead cell staining did not show cytotoxicity in these cultures, indicating that even continuous pulses of supraphysiological NE is not cytotoxic, but can trigger, and perhaps maintain, transdifferentiation of PC cells.

### NE induces characteristic molecular changes involved with NED in PC cells

**Fig. 3:**
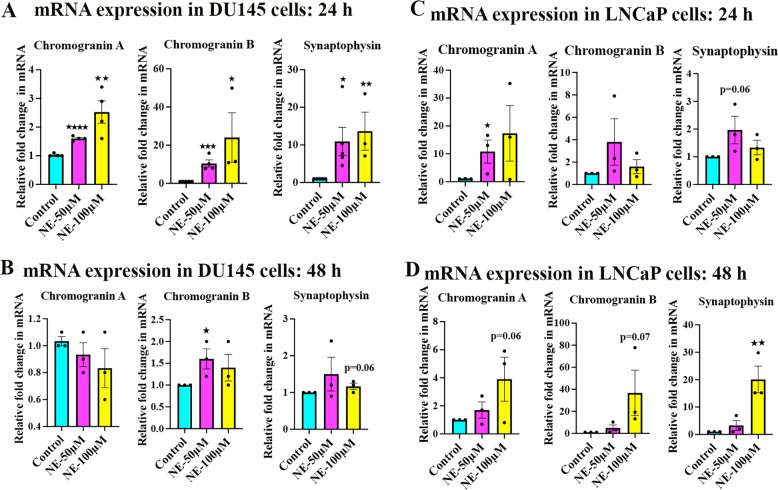
NE induces NED markers in PC cells. **A**, **B** Quantitative real-time PCR shows that NE induces the upregulation of the NED markers CHGA, CHGB, and SYP in DU145 cells at 24 h (**A**) and 48 h (**B**). The data are presented as mean ± SEM (*n* = 3 minimum) and statistically analyzed using standard Student’s *t*-test (unpaired, one-tailed). *p* < 0.05 was considered significant, where *p < 0.05, **p < 0.01, ***p < 0.001, and ****p < 0.0001 compared to control. **C**, **D** Quantitative real-time PCR shows that NE induces the upregulation of the NED markers CHGA, CHGB, and SYP in LNCaP cells at 24 h (**C**) and 48 h (**D**). Data are presented as mean ± SEM (*n* = 3) and statistically analyzed using standard Student’s *t*-test (unpaired, one-tailed). *p* < 0.05 was considered significant, where *p < 0.05 and **p < 0.01 compared to control.

We next asked whether the NED-like morphology induced by NE is true NED by examining the NED-characteristic molecular changes in PC cells. For this, we examined the mRNA expression of the well-known NED markers chromogranin A (CHGA), chromogranin B (CHGB), and synaptophysin (SYP) in DU145 and LNCaP cells after NE treatment. We found a dose-dependent upregulation of CHGA, CHGB, and SYP mRNAs in DU145 cells at 24 h, whereas there was a slight fluctuation in their levels at 48 h (Fig. 3A-B). Strikingly, NE at the lower spectrum of the supraphysiological concentrations tested, such as 50 µM, consistently induced the upregulation of CHGB and SYP at both treatment time points. Similarly, both 50 µM and 100 µM NE induced the upregulation of all three markers in LNCaP cells at 24 h and 48 h, confirming that NE induces NED-characteristic molecular changes in PC cells (Fig. 3C-D).

**Fig. 4:**
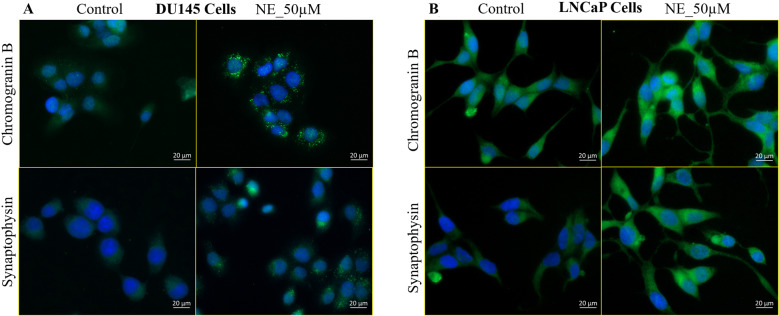
NE induces NED markers in PC cells. **A** Immunostaining shows that 50 µM NE induces the expression of the NED markers CHGB and SYP in DU145 cells at 24 h. Both CHGB and SYP show characteristic granular staining after NE treatment. **B** 50 µM NE induces the expression of CHGB and SYP in LNCaP cells at 24 h. The control cells display weak basal expression of CHGB and SYP, but NE treatment upregulates the expression of these markers, evident by their increased staining intensity. Scale bar, 20 µm.

We then examined the protein expression of CHGB and SYP by immunostaining in PC cells after NE treatment. We observed the characteristic granular staining of both CHGB and SYP in DU145 cells after 50 µM NE. However, they were absent in control cells, indicating that NE specifically upregulates the NED markers expression (Fig. 4A). The LNCaP cells showed mild basal expression of these markers, evident from their uniform cytoplasmic staining in control cells. However, 50 µM NE accentuated their staining intensity in the cells confirming that NE induces the expression of these NED markers (Fig. 4B). Overall, our result indicates that NE, even at a lower spectrum of the supraphysiological concentration, initiates true NED in PC cells.

### Adrβs are involved with NE-mediated NED

We next examined whether Adrβ2 play a role in NE-mediated NED. While 50 µM NE induced the upregulation of CHGB and SYP mRNAs in both DU145 and LNCaP cells indicating initiation of transdifferentiation, pre-treatment with propranolol (an Adrβ1 and Adrβ2 antagonist) inhibited their upregulation (Fig. S5A, B). Similarly, immunostaining showed that propranolol prevents NE-mediated induction of CHGB and SYP remarkably in DU145, whereas its effect was moderate in LNCaP cells (Fig. S5C, D). As our earlier results showed that Adrβ2 has a comparatively higher expression in these cells, the propranolol-mediated inhibition indicates that NE induces NED through activation of Adrβ2 receptors.

### Propranolol inhibits the development and progression of NEPC

**Fig. 5:**
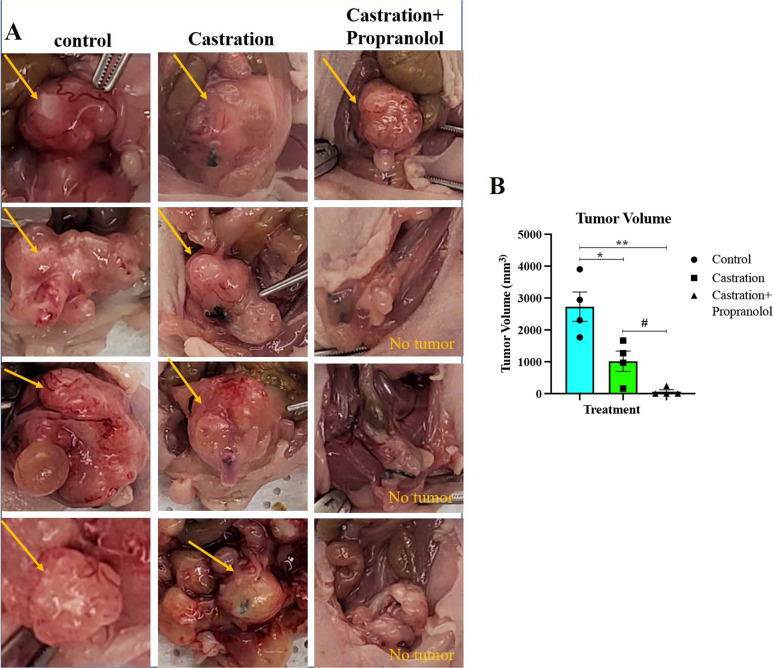
Propranolol inhibits the development and progression of NEPC in orthoptic NEPC models. **A** Individual tumors from the saline control, castration-alone, and castration + propranolol groups show remarkable inhibition of NEPC in the castration + propranolol group. **B** Quantification of “**A**” shows significant reduction in tumor growth in castration + propranolol group compared to the saline control and castration-alone group. Data are presented as mean ± SEM (*n* = 4 animals/group) and statistically analyzed using Standard “*t*” test (unpaired, two-tailed). *P* < 0.05 was considered significant, where *p < 0.05, **p < 0.01 compared to control group and #*p* < 0.05 compared to castration-alone group.

We next examined the effect of Adrβ2 inhibition in NEPC development. We tested this by inhibiting Adrβ2 using propranolol in an orthotopic NEPC model. We used the well-established NEPC cell line, LASCPC-01, to generate the NEPC orthotopic model. Control animals were treated with saline, whereas two other groups were treated with either castration or castration + propranolol. We performed castration to mimic androgen deprivation therapy (ADT), which is a standard treatment approach in the PC clinics prior to advanced stages, such as NEPC. Propranolol treatment lasted for 21 days. Interestingly, we found that all animals in the saline control and castration-alone group developed tumors; however, only one out of the four animals in the castration + propranolol group developed tumor (Fig. 5A, B). Our results thus showed that, while castration alone had a significant effect in lowering the tumor size, its combination with propranolol provides enhanced protection in terms of NEPC development and progression, indicating that Adrβ2 inhibition is an ideal therapeutic strategy for NEPC.

## Discussion

Nerve dependence of PC has gained much attention recently [7, 11, 12]. For example, studies showed that depletion of autonomic nerves suppress PC. Follow-up studies revealed a critical involvement of stromal cells in nerve-dependent PC growth [5]. For instance, selective knockdown of Adrβ2 or Adrβ3 in stromal cells was shown to suppress PC in animal models [5, 6]. Sympathetic nerve-mediated Adrβ2 activation in endothelial cells and resulting enhanced angiogenesis also indirectly promotes nerve-dependent PC growth [6]. Nerves directly alter PC cell dynamics by inducing perineural invasion where PC cells use nerves as physical cues to invade and metastasize [13]. Nerves are also equipped with tumor regulatory machineries, such as active tumor suppressor and DNA repair networks, in modulating tumor dynamics [14–16]. However, whether nerves play a direct role in promoting NEPC has not been studied. In this study, we report the potential role of NE-Adrβ2 axis in inducing NED and NEPC.

A previous study demonstrated that a potent Adrβ2 agonist isoproterenol induces proliferation of PC cells [17]. Therefore, we initially examined whether NE induces PC cell proliferation, but our experiments failed to demonstrate significant cell proliferation in response to 10–30 µM NE, a standard dose used in the literature (data not shown). This led to our attention to a pre-clinical study, which showed that high-grade prostatic intraepithelial neoplasia (HGPIN), an early-stage PC, acquires higher levels of NE compared to healthy prostate [6]. For example, it was shown that mouse HGPIN expresses ~120 ng/mg protein of NE [6]. Inclined to this finding, we and others observed that human prostate tumors possess denser sympathetic innervations [5]. We then estimated that a 40 mg human prostate tumor expresses a total of 4 mg protein. Considering that a complete human prostate tumor weighs a minimum of 1–2 g, the total protein concentration that could be achieved in the tumor is around 100–200 mg, which can consist of 12–24 μg of NE. Then, 100 μM of NE in 1 ml culture offers 16 μg NE, which closely matches the tumor-level, supraphysiological, NE. Interestingly, our experiments using the supraphysiological levels of NEinduced NED in both AR^+^ and AR^−^ PC cells. As NED often leads to NEPC, our finding indicates that aberrant sympathetic activity, and the resulting increased NE signaling, might contribute to NEPC.

Androgen (testosterone) is a major growth factor for PC and ADT is a standard and effective treatment for PC [18]. However, PC eventually develops resistance to ADT, undergoes NED, and emerges as treatment-resistant NEPC. Our findings that supraphysiological NE could induce NED of PC cells suggest that NE might serve as an alternate growth factor for PC, when androgen is deprived, resulting in the transition of PC into NEPC. DU145 cells we used in our experiments lack AR and LNCaP cells, although they express AR, were cultured in androgen-lacking media. Thus, our culture conditions simulated an ADT environment and the effect of NE at these culture conditions in inducing NED supports our argument. We did not, however, examine the effect of NE in inducing NED in androgen enriched conditions.

We found that NE-mediated NED is dependent on Adrβ2. A recent study using a subcutaneous PC xenograft model also demonstrated that Adrβ2 is involved with NED [10]. In general, cutaneous structures have less autonomic innervations compared to internal organs and, hence, the triggering force of Adrβ2 activation in subcutaneous PC models is not clear and less likely to be NE. Therefore, NE being the major catecholamine available in the prostate, our finding of its potential to induce NED warrants special attention. Our experiments showed that propranolol inhibits NE-driven NED. Although propranolol targets both Adrβ1 and Adrβ2, our mRNA analysis showed that Adrβ2 is highly expressed, compared to Adrβ1, in PC cells and prostate tumors, and hence we believe that Adrβ2 might be critical for NE-driven NED.

Overall, our study, for the first time, showed that supraphysiological concentrations of NE facilitates NED of PC cells through activation of Adrβ2. The prostate is supplied by hypogastric and pelvic nerves, providing adrenergic and cholinergic innervations, respectively. The actions of the cholinergic neurotransmitter acetylcholine (Ach) and nerve-derived neurotrophic factors, such as nerve growth factor and brain-derived neurotrophic factor in the prostate milieu might influence the action of NE on PC cells in vivo. Further investigations on the fate of cancer cells in response to the co-ordinated actions of NE, Ach, and neurotrophins may reveal an in-depth understanding of the nature of NE-mediated NED in vivo. Having said that, our in vivo studies provide strong evidence that Adrβ2 inhibition along with castration has superior benefit in managing NEPC. Overall, our findings indicate that NE- Adrβ2 axis is an ideal therapeutic intervention point for NEPC.

## Materials and methods

### Cells, chemicals, and reagents

The PC cell lines DU145 (ATCC HTB81) and LNCaP (ATCC CRL1740) were purchased from ATCC. NE bitartrate (A9512), propranolol hydrochloride (537075) and PI (P4170) were procured from Millipore Sigma. Trizol (15596018), cDNA synthesis kit, Dulbecco’s modified Eagle medium (DMEM)/F12 media (11330032), and fetal bovine serum (12483020) were purchased from Life Technologies. Antibiotic/antimycotic solution (SV3007901) was purchased from HyClone. All other chemicals used were of analytical grade.

### Nerve innervation studies in human prostate tumors

Human tissue studies were performed after receiving approval from the Biomedical Research Ethics Board at the University of Saskatchewan. Fresh, frozen human prostate adenocarcinoma tissues were procured from the Alberta Cancer Research Biobank (ACRB). They were fixed in Zamboni’s fixative overnight at 4 °C and further incubated overnight in 20% sucrose solution. The tissues were then embedded in optimal cutting temperature (OCT) compound and allowed to freeze, followed by 12 µm thick sections taken on slides. The generated sections were blocked for 30 min using 5% donkey serum containing 0.3% Triton X-100. The sections were then co-labeled with primary antibodies against TH (rabbit pAb; AB152, Sigma) and βIII tubulin (chicken pAb; AB9354, Sigma) for 1 h, followed by incubation with a cocktail of anti-rabbit Alexa Fluor® 488 Conjugate (A11034, ThermoFisher Scientific) and anti-chicken Alexa Fluor® 647 (A21449, ThermoFisher Scientific) secondary antibodies for 1 h at room temperature. Sections were then mounted using slow-fade DAPI (S36973, Life Technologies) and images captured using Axio Observer 7 (inverted bright-field/fluorescence microscope, Carl Zeiss, Germany). Quantification of nerve fibers was done manually in a blinded manner by tracing the fibers using Fiji software.

To investigate nerve-cancer cell interface, some sections were also co-labeled with primary antibodies against TH and cytokeratin (mouse mAb; MA1-82041, ThermoFisher Scientific, CA) for 1 h, followed by incubation with a cocktail of anti-rabbit Alexa Fluor® 488 Conjugate and anti-mouse Alexa Fluor® 546 (A21045, ThermoFisher Scientific, CA) secondary antibodies for 1 h at room temperature.

### Morphological assessment of NED features

The PC cells were cultured in DMEM/F12 media containing 10% fetal bovine serum and penicillin and streptomycin cocktail (50 U/ml) at 37 °C and 5% CO_2_ conditions. For the morphological assessment of NED occurrence, 5 × 10^4^ cells/well were seeded in six-well plates and defined treatments were given after overnight incubation. The characteristic NED features, such as compact cell bodies and neurite-like extensions, were then evaluated at 24 and 48 h using Axio Observer 7.

### Cell viability

Cell viability was assessed using both MTT assay (quantitative method) and PI staining (qualitative method). MTT assay was performed as done previously [19]. Briefly, 5 × 10^3^ cells/well were seeded in a 96-well plate and incubated overnight. The cells were then given specific treatments and incubated for 24–96 h. The media was then removed and 1 mg/ml of MTT solution (100 µl) was added to each well followed by 4 h incubation at 37 °C and 5% CO_2_ conditions. After the incubation, the MTT solution was removed and the formazan crystals formed were dissolved in 100 µl of dimethyl sulfoxide. The absorbance of the solution was then read at 570 and 630 nm (for background correction) using SpectraMax M2 (Molecular Devices, San Jose, CA). The percentage change in cell viability was calculated compared to corresponding controls.

For PI staining, 5 × 10^4^ cells/well were seeded in a six-well plate and incubated overnight at 37 °C and 5% CO_2_ conditions. The cells were then treated with varying concentrations of NE for 8, 24, and 48 h, followed by the media removed and 750 µl of freshly prepared PI solution (10 µg/ml) added to the plate. The plates were then incubated at 37 °C for 10 min and the images captured using Axio Observer 7.

### Real-time quantitative reverse-transcription PCR

Total RNA was isolated using TRIzol reagent (15596018, Life Technologies) and quantified using ND-1000 spectrophotometer (NanoDrop Technologies, USA). Then, 500 ng of total RNA was converted into cDNA using a cDNA synthesis kit (4368813, Applied Biosystems) as per the manufacturer’s instructions. The cDNAs were amplified using the specific primers mentioned below, and by using PowerUp™ SYBR™ Green Master Mix (A25741, Applied Biosystems). The cDNA amplifications were done in QuantStudio™ 3 Real-Time PCR System (Applied Biosystems). All reactions were performed in triplicate, and the gene expressions were normalized to the house keeping gene, RPLP.

**Table:** 

Primer	Sequence (5′–3′)
Chromogranin A Forward	GGGATACCGAGGTGATGAAATG
Chromogranin A Reverse	TCTCCTCGGAGTGTCTCAAA
Chromogranin B Forward	GGATGAGGAGGACAAGAGAAAC
Chromogranin B Reverse	CCCTCTCTTCCTCACTTTCTTC
Synaptophysin Forward	CGTGTTTGCCTTCCTCTACT
Synaptophysin Reverse	GCATGGGCCCTTTGTTATTC
Adrβ1 Forward	CAA TGT GCT GGT GAT CG
Adrβ1 Reverse	CCA GGG ACA TGA TGA AGA
Adrβ2 Forward	AGA CCT GCT GTG ACT TCT
Adrβ2 Reverse	CTG AAA GAC CCT GGA GTA GA
Adrβ3 Forward	GCT GGT TGC CCT TCT TT
Adrβ3 Reverse	GCA TAA CCT AGC CAG TTC AG
RPLP Forward	AGCCCAGAACACTGG TCT
RPLP Reverse	ACTCAG GATTTCAATGGTGCC

### Immunofluorescence

NE-treated cells were fixed for 15 min using 4% paraformaldehyde and then blocked for 30 min using 5% donkey serum containing 0.3% Triton X-100. The cells were then incubated with the primary antibodies against CHGB (1 : 100; rabbit; PA5-52605, ThermoFisher Scientific) or SYP (1 : 100; rabbit; MA5-14532, ThermoFisher Scientific) for 3 and 1 h, respectively, followed by incubation with anti-rabbit Alexa Fluor® 488 Conjugate (1 : 100, A11034, ThermoFisher Scientific) secondary antibody for 1 h at room temperature. Cells were then mounted using slow-fade DAPI (S36973, Life Technologies) and examined using Axio Observer 7.

For Adrβ2 and cytokeratin expression in tumor and adjacent normal tissues, the tissues were incubated with primary antibodies against Adrβ2 (1 : 100; rabbit; PA5-14117, ThermoFisher Scientific) and cytokeratin (1 : 100; mouse; MA1-82041; ThermoFisher Scientific) for 1 h at room temperature, followed by incubation with a cocktail of anti-rabbit Alexa Fluor® 488 Conjugate (1 : 100, A11034, ThermoFisher Scientific) and anti-mouse Alexa Fluor® 546 (1 : 100, A21045, ThermoFisher Scientific) for 1 h at room temperature. Cells were then mounted using slow-fade DAPI (S36973, Life Technologies) and examined using Axio Observer 7.

### Western blotting

Total protein from tumor and normal adjacent tissues were isolated using RIPA buffer (Thermo Scientific) containing protease and phosphatase inhibitor cocktail (Thermo Scientific). Thirty micrograms of proteins were then allowed to resolve in an SDS-polyacrylamide gel electrophoresis gel and the resolved proteins were transferred onto a polyvinylidene difluoride membrane. The membrane was then incubated with the primary antibody against Adrβ2 (1 : 1000; rabbit; PA5-14117, ThermoFisher Scientific) for 1 h followed by goat anti-rabbit horseradish peroxidase (HRP) conjugate (1 : 3000; Biorad) and then developed using ECL reagent (Biorad). The membrane was also re-probed with β-actin antibody (1 : 2000; mouse; sc-47778, Santa Cruz Biotechnology) for 1 h followed by goat anti-mouse HRP conjugate (1 : 3000; 170-6516, Biorad) and then developed using ECL reagent. The blots were visualized and images captured, using a Geldoc (Biorad).

### Adrβ2 inhibition studies in vitro

For Adrβ2 inhibition studies, DU145 and LNCaP cells were pre-treated for 1 h with 50 µM propranolol (Adrβ1/Adrβ2 antagonist). The cells were then treated with 50 µM NE either for 6 h (for DU145 mRNA expression studies) or 24 h.

### Orthotopic PC model

All animal experiments were conducted after obtaining approval from the animal ethics committee at the University of Saskatchewan. Adult male athymic nude mice (Crl:NU(NCr)-Foxn1, Charles River, Canada) weighing 20–25 g were used for the study and equal number of animals were randomly allocated into different groups. Tumor induction, treatment, daily monitoring of animals, and final tumor volume reading were done by the same person, with the tumor volume reading done in the presence of an additional person who had no prior knowledge of the experimental layout. The NEPC cells, LASCPC-01, were obtained from ATCC. For castration and orthotopic injection of the cells into the prostate, the mice were first anesthetized with isoflurane and injected with buprenorphine (0.05 mg/kg). A small incision was made in the abdomen, urinary bladder was exposed, and the testicles were located. Castration was performed as described previously [20], and by ligating the testicular arteries followed by removing the testicles by cutting the other end of the arteries along with the attached fat pad. Orthotopic injection was performed by injecting 2 × 10^5^ LASCPC-01 cells in 20 µl (1 : 1 RPMI media and Cultrex basement membrane extract (R&D Systems)) into the exposed prostate. An additional 30 s was provided before removing the syringe from the prostate to minimize cell leaking. The animal’s skin was sutured back and allowed to recover from anesthesia. The animals were weighed once a week. Treatment with either vehicle (saline) or propranolol (20 mg/kg) intraperitoneally was started 7 days after the tumor inoculation procedure. The treatment lasted for 21 days, and then the animals were sacrificed, and tumor sizes recorded.

### Statistics

A minimum of three replicates were performed for each experiment and the exact number of replicates is given in the corresponding figure legend, excluding the samples exempted from the analysis due to technical inaccuracy. Data are presented as mean ± SEM unless otherwise described in the figure legend. The statistical significance was calculated using standard Student’s *t*-test or one-way analysis of variance followed by Dunnett’s post hoc test, wherever appropriate, using GraphPad Prism® 8.0 software. Each group in an experiment is statistically analyzed in similar manner to keep uniform variations between the experimental groups. *P*-value of <0.05 was considered statistically significant.

## Supplementary information

Figure S1

Figure S2

Figure S3

Figure S4

Figure S5

Supplementary figure legends
