# Supplementary figures and images for: Sympathetic signaling facilitates progression of neuroendocrine prostate cancer
Source: Cell Death Discov. 2021 Nov 22;7:364. doi: 10.1038/s41420-021-00752-1 (PMC8608828; doi:10.1038/s41420-021-00752-1)

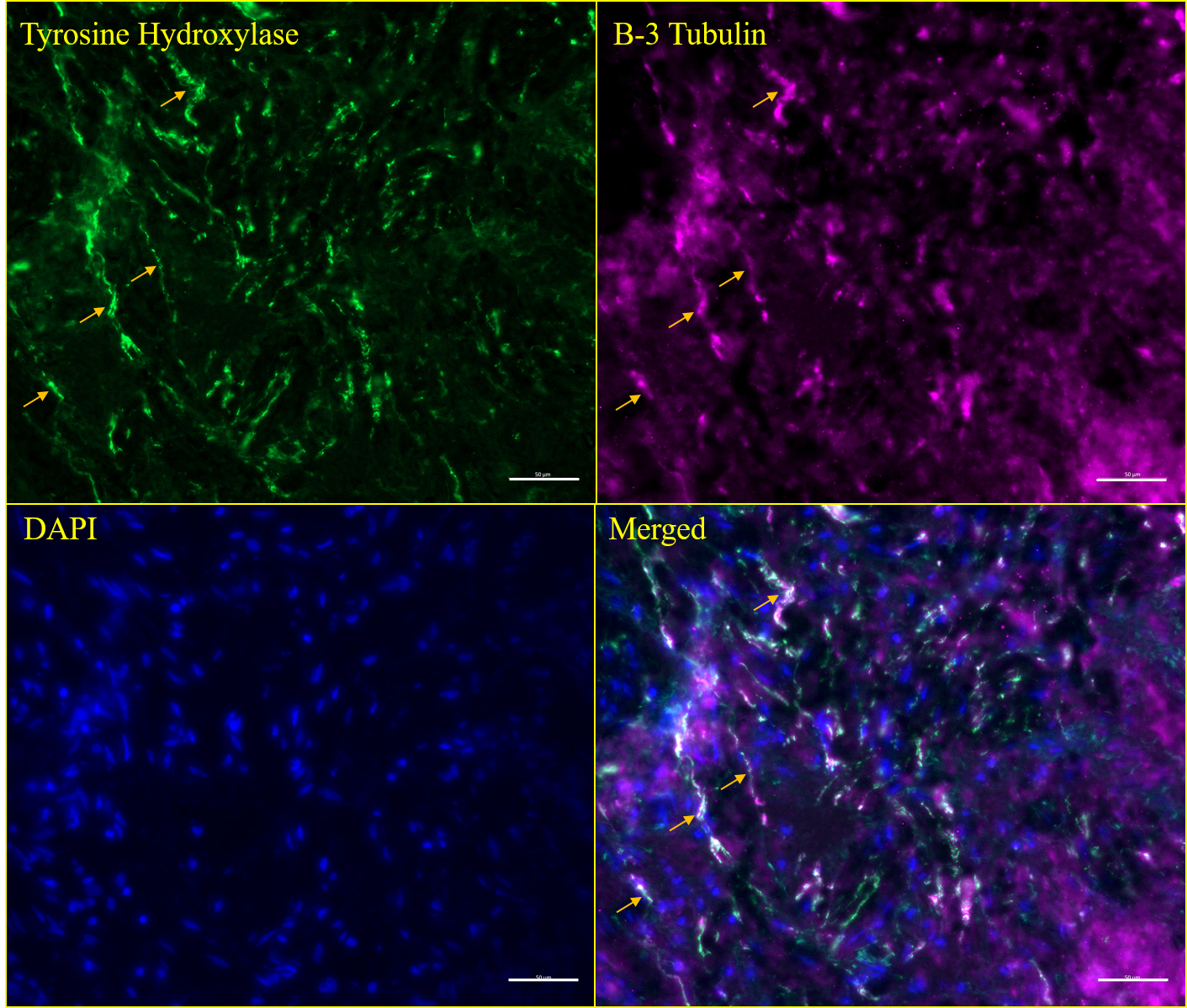

Supplement: Supplementary file 1 — Figure S1 [file 41420_2021_752_MOESM1_ESM.jpg]

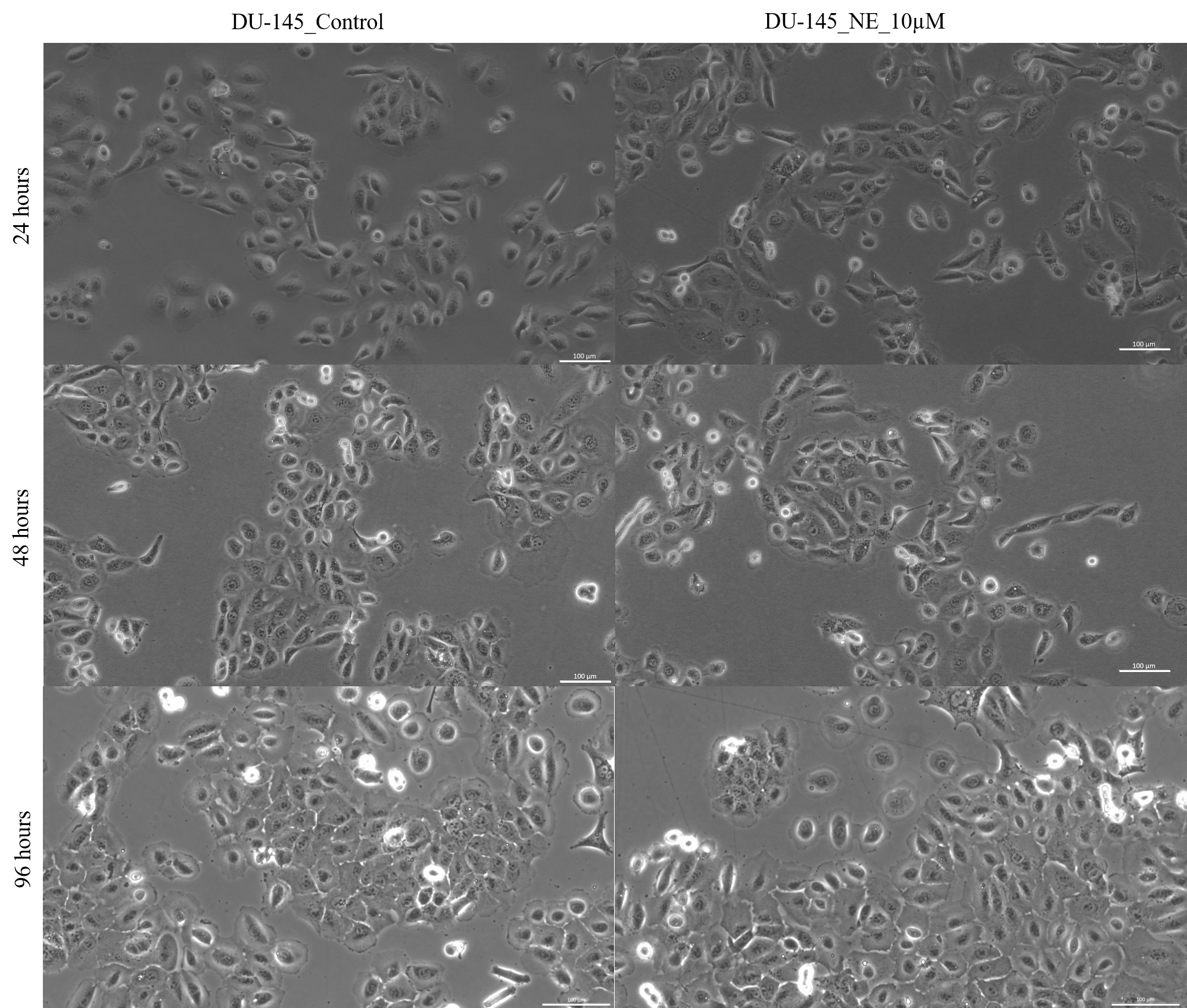

Supplement: Supplementary file 2 — Figure S2 [file 41420_2021_752_MOESM2_ESM.jpg]

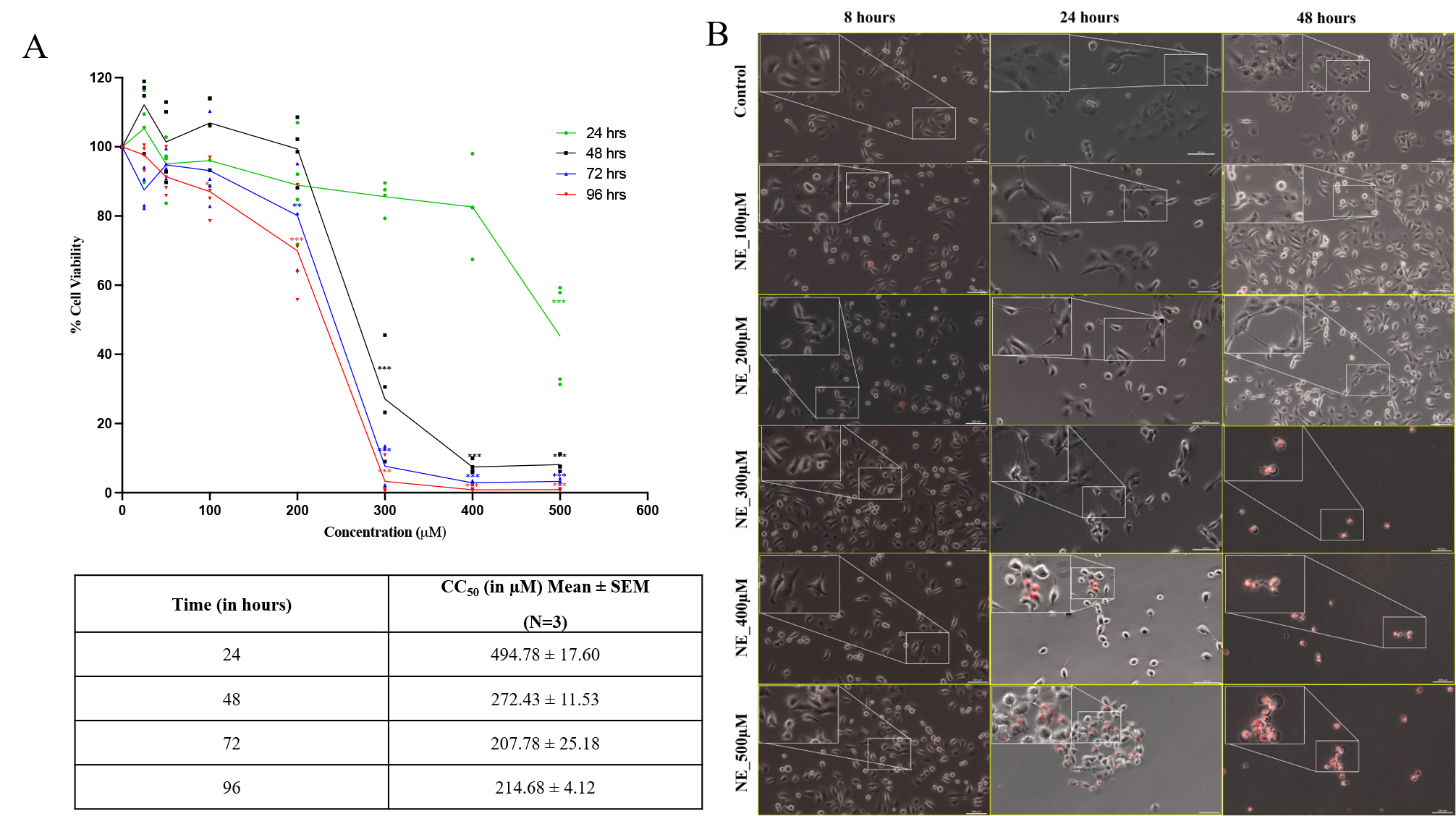

Supplement: Supplementary file 3 — Figure S3 [file 41420_2021_752_MOESM3_ESM.jpg]

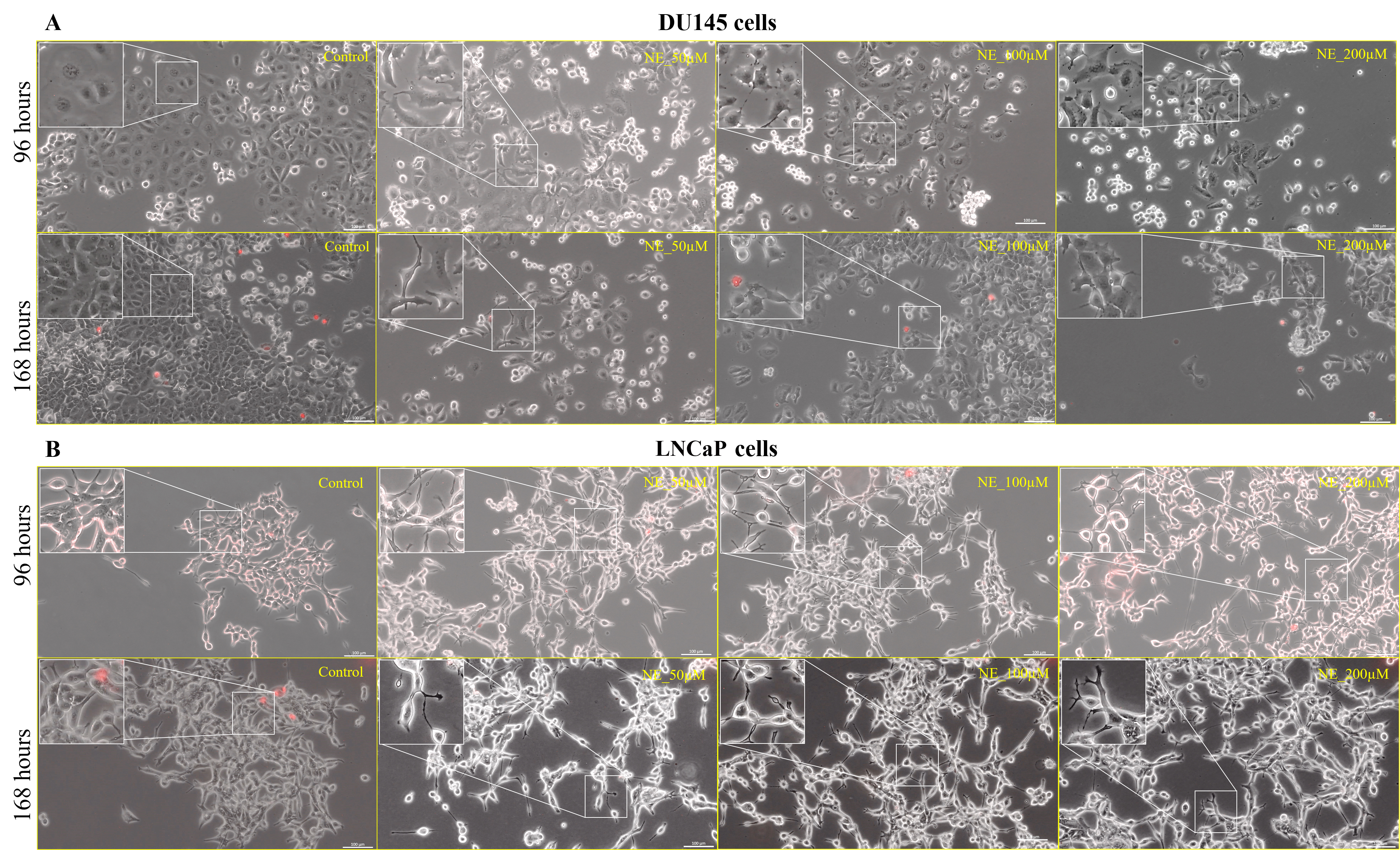

Supplement: Supplementary file 4 — Figure S4 [file 41420_2021_752_MOESM4_ESM.jpg]

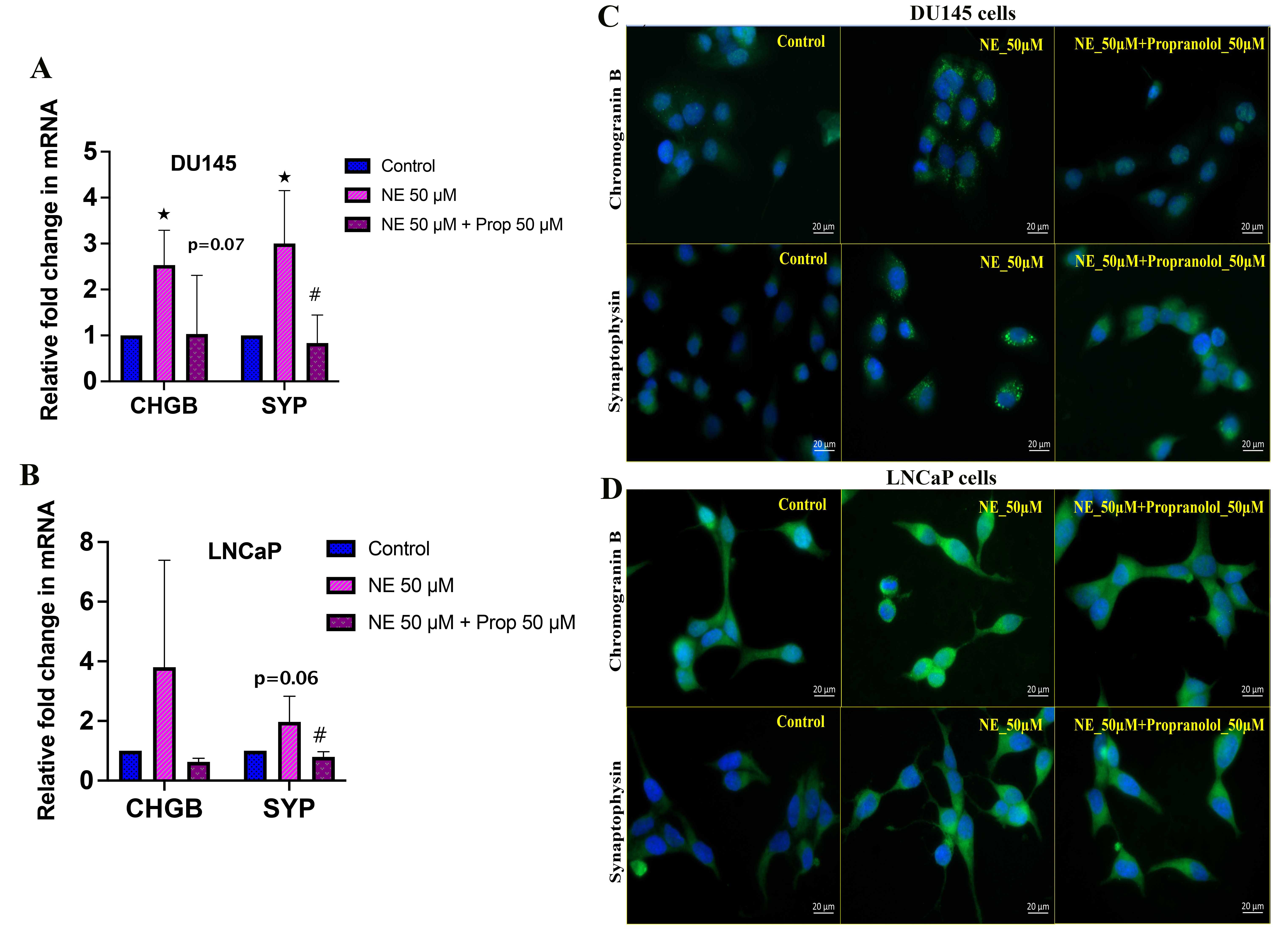

Supplement: Supplementary file 5 — Figure S5 [file 41420_2021_752_MOESM5_ESM.jpg]
