# Supplementary material for: Sympathetic signaling facilitates progression of neuroendocrine prostate cancer
Source: Cell Death Discov. 2021 Nov 22;7:364. doi: 10.1038/s41420-021-00752-1 (PMC8608828; doi:10.1038/s41420-021-00752-1)
Supplement: Supplementary file 6 — Supplementary figure legends [file 41420_2021_752_MOESM6_ESM.docx]

**Supplementary figure legends**

**Figure S1:** **Co-staining of tyrosine hydroxylase (TH) and βIII tubulin in sympathetic nerves:**

Dual staining of TH (green) and the pan-neuronal marker βIII tubulin (pink) in a human prostate tumor shows that TH specifically stains nerve fibres, indicated by the arrows in the merged image. Scale bar, 50 µm.

**Figure S2: 10µM NE does not induce NED-related morphological changes in DU145 cells.** Bright field images of DU145 cells after 10µM NE treatment for 24 h-96 h. No remarkable NED-related morphological changes were observed in these cells compared to control. Scale bar, 100 µm.

**Figure S3: Effect of NE on cell viability.** A) MTT assay shows % cell viability of NE treated DU145 cells. Data in the graph presented as individual replicates (n = minimum 3) with mean or median connected and were statistically analysed using One-way ANOVA followed by Dunnett’s post hoc test. P<0.05 was considered significant, where ***p<0.001 **p<0.01 and *p<0.05, compared to control. The CC_50_ values (n= minimum 3) of NE at various time points are provided below. B) [Related to Figure 2] Representative merged images (bright field and propidium iodide staining (red)) show that NE up to 200 µM concentration is not cytotoxic, evident by lack of PI staining. However, NE at concentrations 300 µM and above induces cytotoxicity. Scale bar, 100 µm. [The corresponding bright field alone versions of some of these images are also presented in figure 2].

**Figure S4: Pulsed exposure of up to 200µM NE is not cytotoxic but induces NED.** A-B) Representative merged images (bright field and propidium iodide (PI) staining (red)) show that NE, at the shown doses, does not induce cytotoxicity in DU145 (A) or LNCaP (B) cells, evident from the lack of PI staining in most of the cells. However, NE induces NED like morphological changes (neurite-like outgrowth and compact cell bodies: see insets) in these cells. Scale bar, 100 µm.

**Figure S5: Effect of Adrβ2 inhibition on NE-induced NED. A)** Quantitative real-time PCR data shows that propranolol inhibits NE-mediated upregulation of CHGB and SYP in DU145 cells at 6 h. Data are presented as mean ± SEM (*n* = 3) and statistically analyzed using Standard “*t*” test (unpaired, one-tailed). *p* < 0.05 was considered significant, where **p* < 0.05 compared to control group, *p* = 0.07 compared to NE 50 µM group and #*p* < 0.05 compared to NE 50 µM group. **B)** [Related to figure 3C with additional data of propranolol compared with the same controls and NE 50µM treatments presented in figure 3C, as shown here]. Quantitative real-time PCR data also shows that propranolol inhibits NE-mediated upregulation of CHGB and SYP in LNCaP cells at 24 h. Data are presented as mean ± SEM (*n* = 3) and statistically analyzed using Standard ‘*t*’ test (unpaired, one-tailed). *p* < 0.05 was considered significant, where *p* = 0.06 compared to control group and #*p* < 0.05 compared to NE 50 µM group. **C**, **D** [Related to Figure 4 A-B with additional data of propranolol compared with the same controls of DU145/CHGB & LNCaP/SYP presented in Figure 4A-B, as shown here]. Immunostaining shows that propranolol inhibits NE-driven induction of CHGB (green) and SYP (green) remarkably in DU145 cells (**C**) and moderately in LNCaP cells (**D**) at 24 h. Scale bar, 20 µm.
